# Improvement of Ferulic Acid Antioxidant Activity by Multiple Emulsions: *In Vitro* and *In Vivo* Evaluation

**DOI:** 10.3390/nano11020425

**Published:** 2021-02-08

**Authors:** Antonia Mancuso, Maria Chiara Cristiano, Rosanthony Pandolfo, Manfredi Greco, Massimo Fresta, Donatella Paolino

**Affiliations:** 1 Department of Health Sciences, University “Magna Græcia” of Catanzaro, Campus Universitario “S. Venuta”, 88100 Catanzaro, Italy; antonia.mancuso@unicz.it (A.M.); rosantony.pandolfo@unicz.it (R.P.); fresta@unicz.it (M.F.); 2 Department of Experimental and Clinical Medicine, University “Magna Græcia” of Catanzaro, Campus Universitario “S. Venuta”, 88100 Catanzaro, Italy; mchiara.cristiano@unicz.it; 3 Plastic Surgery Unit, Department of Clinical and Experimental Medicine, “Magna Graecia” University, 88100 Catanzaro, Italy; manfredigreco@unicz.it

**Keywords:** multiple emulsion, ferulic acid, natural compound, antioxidant, *in vitro*, *in vivo*, skin delivery

## Abstract

Ferulic acid is a derivative of cinnamic acid showing efficacious anti-oxidant activity. It catalyzes the stable phenoxy radical formation, upon absorption of ultraviolet light, giving the strength to ferulic acid for terminating free radical chain reactions. Ultraviolet rays are one of the most dangerous factors that daily assault the skin, causing excessive generation of reactive oxygen species (ROS), which are regarded to be important contributors to a variety of cutaneous alterations. The skin possesses endogenous antioxidant defense systems, but the excess of ROS leads to an oxidant–antioxidant imbalance. Although ferulic acid is daily introduced in human organism with the diet, its bioavailability after oral administration is poor, particularly in the skin. The aim of this investigation was to evaluate three types of emulsions (W/O/W multiple emulsions and two simple emulsions) as suitable formulations for topical application of the active compound. *In vitro* studies were performed to investigate the stability and release profiles of these systems. Multiple emulsions showed great stability and the best ability to carry and release ferulic acid. *In vivo* evaluations highlighted their best capability to treat UV-B-induced erythema. These findings suggested multiple emulsions as an innovative and more efficient vehicle for topical application of ferulic acid.

## 1. Introduction

In the last decades, natural products containing antioxidants, such as phenolic compounds, polyphenols and flavonoids, are object of the researchers’ interest due to their potential therapeutic effects [1,2,3]. In particular, phenolic compounds have been proposed for the treatment of inflammatory diseases, cancer, obesity, diabet, allergic reactions or to prevent/counteract neurodegenerative disorders [4,5,6,7]. Ferulic acid (4-hydroxy-3-methoxycinnamic acid), a derivative of cinnamic acid, arising from the metabolism of phenylalanine and tyrosine, is ubiquitous plant constituent [8] that shows an efficacious anti-oxidant activity due to the phenolic ring and unsaturated side chain, which can easily produce a resonance stabilized phenoxy radical, characterized by a potent antioxidant activity. The reactive radical that collides with ferulic acid, easily abstracts a hydrogen atom to form the phenoxy radical that is highly resonance stabilized since the unpaired electron may stay on the oxygen or be delocalized across the entire molecule. The extended conjugation in the unsaturated side chain also stabilizes the phenoxy radical [9]. Cause of its incapacity to initiate or propagate a radical chain reaction, the phenoxy radical ends its life in a collision and condensation with another ferulate radical, leading to the formation of the dimer curcumin, which presents a second phenolic hydroxyl group able to further improve the radical scavenger activity due to the greater resonance stabilization and o-quinone formation. Finally, the ability of the ferulic acid to strengthen scavenger radical enzymes and to inhibit enzymes that catalyze the formation of free radicals, makes it an excellent scavenger of free radicals [10,11,12].

Ultraviolet rays often cause the excessive generation of reactive oxygen species (ROS). UV solar spectrum is divided into three segments based on the wavelengths of the radiation: Short wave (UV-C; 200–290 nm, fortunately are absorbed by the atmospheric ozone layer and normally doesn’t reach the surface of the hearth, because they possess enormous energy and can damage DNA molecules); mid wave (UVB; 290–320 nm, approximately 5% of the total solar UV radiation and mainly responsible for a variety of skin diseases, are able to penetrate the skin to a depth of approximately 160–180 μm) and long wave (UV-A; 320–400 nm, penetrates deeper into the epidermis and dermis of the skin, and the extensive exposure induce the generation of singlet oxygen and hydroxyl-free radicals, that may cause damage to cellular macromolecules, such as proteins, lipids and DNA) [13,14]. The interaction of the radiations, at about 345 nm, with trans-urocanic acid in skin, generate single oxygen molecules. This is the main cause of photodamage, an alteration in the dermal structure of extracellular matrix (ECM) that elicits oxidative stress changes such as connective tissue alterations, skin damage and increased epidermal thickness [15]. As stated above, the main causes of these dangerous effects are ROS that are reactive molecules, such as peroxides, superoxide, hydroxyl radical, and singlet oxygen [16].

Skin possesses an elaborate endogenous protective system consisting of non-enzymatic and enzymatic components such as vitamin E, vitamin C, catalase, superoxide dismutase, glutathione peroxidase, mostly expressed in extracellular space and in the epidermis and dermis layers [17]. However, since the increase of ROS expression is matched with the overproduction of nitric oxide from keratinocytes, this often leads to the impairment in antioxidant defenses [18]. This is one of the most important factors that induce skin cutaneous alterations (i.e., erythema, sunburn cells, hyperplasia, hyperpigmentation and premature skin aging) or pathologies as inflammation, immunosuppression, and photo-carcinogenesis [19].

The topical administration of antioxidant agents as phenolic structures, and in particular of ferulic acid, may represent a valid tool to improve the amount of endogenous antioxidants and to reduce the photodamage caused by UV radiations. Even if ferulic acid is introduced daily in human organisms with the diet, its bioavailability after oral administration is poor, particularly in the skin district. The topical administration of ferulic acid, by mean of a suitable formulation could ensure an efficacious activity against UV-induced photodamage [20].

Around the topical formulations, multiple emulsions are a complex kind of emulsive system that may be considered as “emulsions of emulsions” [21]. To date, the most known multiple emulsions are water-in-oil-in-water (W/O/W) and oil-in-water-in-oil (O/W/O), where the continuous phase is respectively water and oil. The methods of preparation of multiple emulsions largely reported in literature are a phase inversion method, a one-stage emulsification method and a two-stage emulsification method [22,23]. These emulsive systems present significant potentialities for the application of pharmaceutical and cosmetic active compounds because they are characterized by prolonged release of drugs, the possibility to combine incompatible substances in one vehicle and/or the protection of unstable molecules [24,25,26].

Since ferulic acid is a potent photoprotective ingredient, the aim of this work was to compare three types of ferulic acid-loaded emulsions (one step-multiple emulsions, O/W and W/O single emulsions), performing *in vitro* and *in vivo* studies, as suitable formulations for topical treatment of oxidative stress. The stability of these formulations was evaluated and their effectiveness to deliver ferulic acid to treat UVB-induced erythema was assessed. Among the analyzed formulations, multiple emulsions showed the best stability profile and great ability to carry and release ferulic acid.

## 2. Materials and Methods

### 2.1. Materials

Arlacel P135 (PEG-30 dipolyhydroxystearate), Arlamol E (PPG-15-stearyl ether), Arlamol HD (isoexadecane), Brij 72 (steareth 2), Brij 721 (steareth 21), Estol 3603 (caprylic/capric trygliceride) and Symperonic PE F/127 (poloxamer 407) were purchased from Bregaglio S.r.l. (Milan, Italy). Cetyl alcohol, Dimethicone, Glycerin, Stearic acid and Triethanolamine were purchased from Carlo Erba (Milan, Italy). Carbomer was purchased from Lubrizol Italia S.r.l. (Milan, Italy). Ferulic acid was purchased from Sigma Aldrich (Milan, Italy).

### 2.2. Preparation of Emulsions

Three different emulsions were prepared: A water-in-oil (W/O) emulsion; an oil-in-water (O/W) emulsion; a multiple emulsion (ME) obtained using a ‘one-step’ procedure. The same amount of ferulic acid (0.2% *w*/*v*) was added during the preparation of emulsions.

Formulations were realized as previously reported by our research group [27] and as shown in Table 1. For the preparation of the W/O emulsion, the components of aqueous phase containing the active compound and the components of oily phase were solubilized in two different beakers and heated by using a magnetic heating plate up to the temperature of 75 °C. When all components have been dissolved, the aqueous phase containing the active compound has been carefully added to the oily phase enriched with a lipophilic emulsifier, Arlacel P135, using a Silverson Mixer Homogenizer (Crami Group S.r.l., Milan, Italy) at a constant stirring of 7500 rpm/min for 10 min. Formulations were finally stirred at 4500 rpm/min until the emulsion has spontaneously reached room temperature.

Similar to W/O emulsion, the O/W emulsion was performed adding the oily phase to the aqueous phase, containing a hydrophilic emulsifier, Poloxamer 407, using a constant stirring of 4500 rpm/min (both phases were at 70 °C) and maintaining the stirring until the emulsions were at 25 °C.

The ME was obtained adding the oily phase, including the lipophilic emulsifier, to the aqueous phase (both phases at 70 °C), under a constant stirring at 4500 rpm/min, until the emulsion reached the room temperature (~25 °C). The mixture of surfactant agents, Brij 72 and Brij 721 as compounds that have the characteristic to create spontaneously a multiple emulsion, allows to obtain the final formulation.

### 2.3. Chemical-Physical Characterization of Emulsions:

#### 2.3.1. Morphological Analysis by Optical Microscopy

The Microscopy Axiophot Zeiss (Carl Zeiss, Oberkochen, Germany) equipped with a lamp 12 V/100 W, a 100×/1.30 Plan-Neofluar lens, a ×100 ocular and an Axiocam 105 color camera, was used for characterization studies [27]. This optical microscopy has permitted to analyze the actual realization of the desidered formulation, the mean size and the distribution size of the various emulsive globules of the sample. The samples were investigated in transmitted light, bright field mode. The AxioVision Software 4.6 (Carl Zeiss, Oberkochen, Germany) was used to analyze the images.

#### 2.3.2. Stability, Homogeneity of the Samples and Diameter Kinetic Profiles

Turbiscan Lab^®^ Expert (Formulaction, L’Union, France) analysis was performed to investigate the long-term stability of emulsions [28]. The instrument is equipped with a pulsed near infrared LED, set at a wavelength of 880 nm and it records the light that is transmitted (T) and backscattered (BS) through the whole height of the sample. In detail, each analyzed formulation was poured in a glass tube and any variation of the backscattered (∆BS) and/or transmitted (∆T) signals, was detected as a function of two reference standard that are a silicon oil and a latex suspension to investigate the volume fraction (migration) and diameter (coalescence) changes of emulsions [29]. Measurements were collected up to 3 h, at the temperature of 24 ± 1 °C.

The Turbiscan Lab^®^ apparatus (Formulaction, L’Union, France) was also used to investigate diameter kinetic profile over time (3 h) [30]. Any variation of average sizes related with globules of sample was recorded and reported as a function of time. Experiment has been performed in triplicate and data reported are representative of results collected from the independent experiments.

Moreover, the globule size distribution and homogeneity of emulsions samples were given by dynamic laser light scattering technique carried out with a Mastersizer 2000 (Malvern Instruments, Worcestershire, UK), a particle size analyzer. The analysis was carried out at a 90° scattering angle. In order to produce a required count rate (100–500 kcps), the sample was freshly diluted before analysis and the samples were analyzed 24 h after their preparation.

### 2.4. In Vitro Release of Ferulic Acid

Static Franz diffusion cells system (Laboratory Glass Apparatus, CA, USA) provided to measure the release of ferulic acid from simple and multiple emulsions, using cellulose acetate membranes having molecular cutoff of 3 kDa (Spectrum Laboratories Inc., Eindhoven, The Netherlands), with a diffusion surface area of 0.75 cm^2^, and a receptor volume of 4.5 mL [31]. The membranes were horizontally positioned between the donor and the receptor compartment of Franz cells. The receptor compartment was filled with isotonic pH 7.4 phosphate buffer [20] and it was continuously stirred with a small magnetic stirring bar for maintaining its homogeneity. The Franz cells were thermostated with a thermostatic bath GR 150 (Grant, Cambridge, UK) at 35 °C. After the moistening of membrane surface with receptor phase, 200 µL of each formulation were added to the donor compartment at the same temperature. During 10 h, at prefixed time intervals, 200 μL of receptor phase were collected, and replaced with the same volume of receptor phase. Collected samples were analyzed by HPLC. Each experiment was performed in triplicate and data were reported as mean values ± standard deviations.

### 2.5. HPLC Determination of Ferulic Acid

HPLC analysis were performed using a Jasco Europe HPLC system equipped with a 20 μL Rheodyne model 7125 injection valve (Rheodyne, Cotati, CA) a Jasco MD 1510 multiwavelength UV detector, LC net II interface and Chromnav software 2.0 (Jasco Europe, Milan, Italy). The reverse phase consisted of Nucleosil 100-5 C18 column (250 × 4.6 mm internal diameter, 5 μm particle size) (Applied Biosystems, Foster City, CA, USA). The mobile phase 1consisted of acetonitrile:water (19:81 *v*/*v*) containing 2% acetic acid, the flow-rate was set at 1.0 mL/min. Ferulic acid detection was performed at *λ_max_* = 302 nm [20]. Before the injection, samples were filtered by a Millex HV13 filter (Waters-Millipore Corporation, Milford, UK). To quantify the ferulic acid it was used an external standard curve in the linear concentration range between 0.1 and 10 μg/mL by a stock of a standard aqueous solution of ferulic acid (1 mg/mL), having a *r*^2^ value of 0.9999. The amount of ferulic acid was obtained according to the following equation,

AUC = 0.5437*x* + 0.0103 (1)

where *x* was the concentration (μg/mL) of the active compound.

### 2.6. Percutaneous Membranes Permeation through Human Stratum Corneum and Epidermis (SCE)

#### 2.6.1. Isolation of SCE-Membranes

To verify the percutaneous permeation of the formulations, we tested the three kinds of emulsions on human stratum corneum and viable epidermis membranes (SCE-membranes).

The permeation experiments were performed using dynamic Franz-type diffusion flow cell systems (Laboratory Glass Apparatus, Berkeley, CA, USA), having a surface area of 0.75 cm^2^ and a flow-through receptor compartment of 4.5 mL. The stratum corneum upper side of SCE-membranes was assembled between the donor and the receptor compartments and experiments were performed under occlusive conditions. To ensure the receptor solution homogeneity was used a Variomag^®^ Multipoint stirrer (Daytona Beach, FL, USA) equipped with small magnetic bars (700 rpm). Sink conditions were guaranteed by a degassed pH 7.4 isotonic phosphate buffer pumped by Minipuls 3 peristaltic pump (Gilson Italia S.r.l., Milan, Italy) at a flow rate of 2 mL/h. A fraction collector FC 204 (Gilson Italia S.r.l., Milan, Italy) was added to the dynamic Franz-type diffusion flow cell apparatus to collect the receptor solution as a function of time.

Bioptic samples used for the analysis were obtained from abdominal reduction surgery performed on human patients with mean age of 35 ± 8 years. The study was performed in accordance with the Declaration of Helsinki, and the protocol was approved by the Research Ethics Committee of the “Magna Graecia” University of Catanzaro (Approval number: 390/2019).

Briefly, the subcutaneous fat tissue was removed as previously reported [32] and the SCE membranes isolation from the dermis were performed putting the skin specimens in a water bath warmed to 60.0 ± 1.0 °C for 2 min. SCE membranes were scratched by a surgery scalpel and then were put in a desiccator containing phosphoric anhydride as a drying agent, to dehydrate. Finally, the membranes were stored at 4.0 ± 1.0 °C until their use in aluminum foils. For these experiments the SCE membranes were re-hydrated at room temperature for 1 h before they were interposed between the donor and the receptor compartment of the Franz cells.

#### 2.6.2. Percutaneous Permeation Studies

Percutaneous permeation experiments were performed using Franz cells, administering multiple emulsion containing ferulic acid (0.2% *w*/*v*) on skin sections and comparing them with the O/W and W/O emulsions containing the same amount of active. Briefly, 200 μL of each formulation were added in the donor compartment at a thermostated temperature of 35 ± 0.1 °C (GR 150 thermostat). Samples of the receptor phase were collected hourly until 10 h and the withdrawn volume was replaced at picked times. Finally HPLC analysis gave the results of the amount of ferulic acid permeated through the SCE membranes, expressed as the mean value of six different experiments ± the standard deviation.

To calculate the *in vitro* percutaneous fluxes (µg/cm^2^·h^−1^) of ferulic acid, the amount of ferulic acid permeated through SCE membranes was plotted with time and the slope of the linear portion of the curve (steady-state) was divided by the area of the SCE membrane surface [33].

### 2.7. In Vivo Tolerability Studies

The *in vivo* applicability of emulsions containing ferulic acid was evaluated using a non-invasive method [34]. The study involved 12 healthy human volunteers (both sexes) with a mean age of 31 ± 5 years, who provided their written and informed consent. All subjects which participated to the study had skin types II and III. They were housed and acclimated 30 min before the experiments at room conditions (22 ± 2 °C, 40–50% R.h.). The reflectance spectrophotometer X-Rite SP60 (X-Rite Incorporated, Grandville, Michigan, MI, USA) was used to detect any change in the skin color following the administration of the empty formulations. In detail, the instrument was calibrated, before the beginning of any experiment, with the provided white standard traceable to the National Bureau of Standard’s perfect white diffuser. A specific Spectrostart program (X-Rite Incorporated, Grandville, Michigan, MI, USA) performed all the colour calculations from the spectral data obtained. Reflectance spectra were recorded within the wavelength range of 400–700 nm using illuminant C and 2° standard observer.

The following experimental protocol was used: four sites were demarcated (1 cm^2^ in diameter) on the ventral surface of the forearm of each volunteer; measurements were recorded for each site before the administration of samples (baseline); 200 µL of empty formulations (ME, W/O emulsion and O/W emulsion) were applied on the respective skin site, leaving at least 2 cm between sites to avoid contaminations; the fourth site was treated with saline solution (0.9% NaCl *w*/*v*), that was used as negative control. Before the values were recorded, each site was cleaned to remove any residue of the applied formulation. The values of erythema index (E.I.) were monitored and recorded following 6 h, 24 h, 48 h and 72 h of treatment, according to the following equation,

(2) $$E.I.=\;100\;[\log\frac{1}{R560}+1.5(\log\frac{1}{R540}+\log\frac{1}{R580})-\;2(\log\frac{1}{R510}+\log\frac{1}{R610})],$$

where R was the reflectance at different wavelengths. In detail, 540, 560 and 580 were the absorption peaks of hemoglobin, whilst 510 and 610 were those referred to melanin.

The variations of erythema index values (ΔE.I.) were calculated subtracting the baseline values from the values of E.I. recorded at the respective site, as a function of time.

Studies involving human subjects were carried out in accordance with the Declaration of Helsinki guidelines. The protocol was approved by the Research Ethics Committee of the “Magna Graecia” University of Catanzaro (Ethics approval numbers: 390/2019; 392/2019).

### 2.8. In Vivo Evaluations of the Photoprotective Effect of Ferulic Acid

The efficacy against the photodamage of the different types of emulsions (W/O, O/W emulsions and ME) containing ferulic acid, was investigated *in vivo* on healthy human volunteers as previously reported [20]. In detail, we evaluated the ability of samples to reduce the physically-induced erythema using the X-Rite SP60. The minimal erythema dose (MED) was investigated for each person and then the subject was exposed to a double irradiating dose with respect to the MED. The skin erythema was induced using the ultraviolet lamp UVM-57 (UVP, San Gabriel, CA, USA), which provided emission in a range between 290–320 nm with an output peak of 302 nm. The flux rate measured at the skin surface was 0.80 mW∙cm^−2^. The reflectance visible spectrophotometer, SP60 (X-Rite Incorporated, Grandville, Michigan, MI, USA), having 0° illumination and 45° viewing angle, was calibrated with a supplied white standard traceable to the National Bureau of Standard’s perfect white diffuser and used for monitoring UVB induced erythema. A personal computer, connected to the instrument, carried out all color calculations from the spectral data. Reflectance spectra were acquired over the wavelength range of 400–700 nm by using illuminant C and 2° standard observer.

For each subject, 200 µL of various formulations, containing ferulic acid (FA) were applied on demarcated sites (1 cm^2^). Eight sites were defined on the forearm of each healthy volunteer and two sets of experiments were performed. In the first set, the skin was previously irradiated and then 200 µL of each emulsion were applied to irradiated sites, using Hill Top Chambers (Hill Top Research Inc., Cincinnati, OH, USA) for three hours. Two sites were left untreated after the UV exposition, and were used as control. A distance of at least 2 cm was left between sites to avoid any interference. Experiments were performed on 12 healthy human volunteers. The erythema was induced before the administration of each sample and monitored, through reflectance spectrophotometry for 72 h, until it disappeared.

In the second set of experiments, a pre-treatment approach was used to further investigate the photoprotective effect of emulsions. Briefly, 200 µL of each formulation were applied on the marked sites of the forearm of each volunteer for two different times (1 h and 5 h). At the end of the scheduled times, the treated skin surface was washed to remove any residue and the erythema was induced. Finally, the induced erythema was monitored using reflectance spectrophotometric readings until 72 h. Erythema index values were calculated using the Equation (2) (see Section 2.7). The E.I. baseline values were taken at each designated site before UVB irradiation and were subtracted from the E.I. values obtained at each time point, to determine ∆E.I. values following UVB exposure.

### 2.9. Statistical Data Analysis

Statistical data analysis was carried out using a one-way ANOVA. The Bonferroni’s *t*-test was used to check the ANOVA test. Data with *p* value ≤ 0.05 are considered statistically significant.

## 3. Results and Discussions

### 3.1. Preparation and Chemical-Physical Characterization of Emulsions

Three types of emulsion formulations were prepared for this study, i.e., two simple emulsions (W/O and O/W) and a one step multiple emulsion (ME). Once prepared, each emulsion formulation appeared as a white semisolid preparation which showed no macroscopic phase separation at room temperature. Since stability is the main challenge for emulsions, further investigations were performed and, following the preparation of samples, their physico-chemical, morphological and technological parameters were investigated.

**Figure 1:**
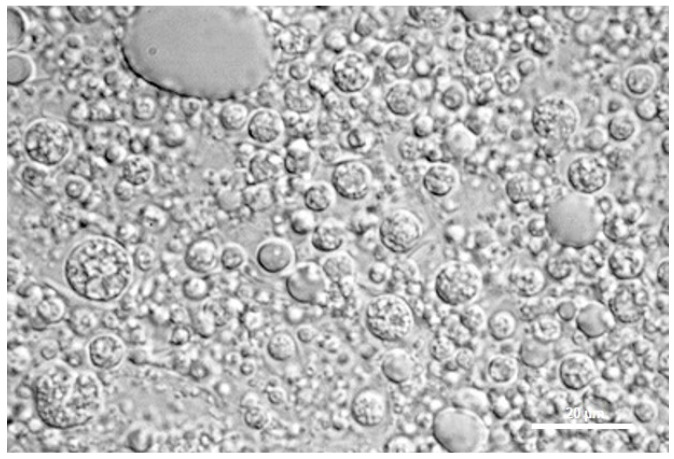
Multiple emulsions after 6 h from their preparation, a photomicrograph obtained by optical microscopy. Scale bar represents 20 µm.

Optical microscopy studies allowed to investigate also the inner structure of ME (Figure 1) and simple emulsions (Figure S1). ME appeared as three-phases systems with a reservoir characteristic, having an external aqueous phase with dispersed oily emulsive globules which in turn contain several smaller internal water globules. Figure 1 reported the multiplicity of the droplets of ME, having mean size of the oil droplets of 6 ± 2 µm and mean size of the inner aqueous droplets of ~1 µm.

**Figure 2:**
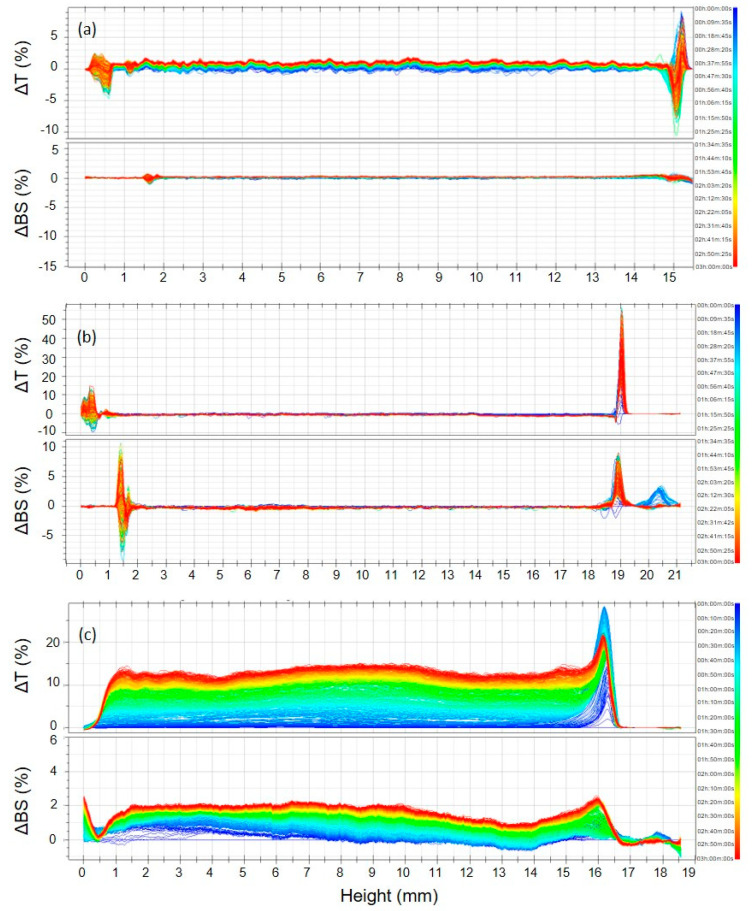
Delta Transmission (ΔT) and delta Backscattering (ΔBS) profiles of (**a**) ME, (**b**) O/W emulsion and (**c**) W/O emulsion determined by Turbiscan Lab^®^ Expert. Various runs are representative of three independent experiments. Data are reported as a function of time (0–3 h) and sample height (from 2 to 8 mm). (The numbers in the axes have been enlarged to improve the visibility of results.)

Since one of the main limits of emulsions is the appearance of creaming or coalescence phenomena, the stability of samples has been investigated through Turbiscan Lab^®^ analysis and it was measured as a function of the Δbackscattering profiles (Figure 2). The stability profiles recorded for the three formulations were within acceptable ranges of values. In fact, as previously reported [29], no changes in globule size occurred in the case of formulations having variation in backscattering profiles (ΔBS) within ± 2%, whilst variation over 10% are representative of unstable samples. However, as can be seen in Figure 2, ME (panel a) and O/W emulsions (panel b) were more stable than W/O emulsions (panel c). Several other studies confirmed the lower stability of W/O emulsions compared to O/W once and this is probably related to the high mobility of water droplets in the oil phase. However, sometimes the kinetic transition leading to the separation of water from oil phases could be so slow to consider these emulsions as metastables [35,36] and this is the case of our W/O sample.

The greater stability recorded from ME is probably related to its composition, involving stabilizing compounds, such as Brij 72 and Brij 721, that are arranged at the oil-water interface [37,38]. On the other hand, the inorganic salt MgSO_4_, that is a constituent of O/W emulsions, could interact with the surfactant layer at the oil-water interface improving the emulsion stability [39]. Indeed, ME and O/W emulsions showed values of backscattering of ~0.5% for the whole height of the sample, thus suggesting that these formulations were not subject to creaming or sedimentation phenomena. Instead, W/O emulsions showed more significant variations in its ΔBS profiles as demonstration of their lesser stability over time.

**Figure 3:**
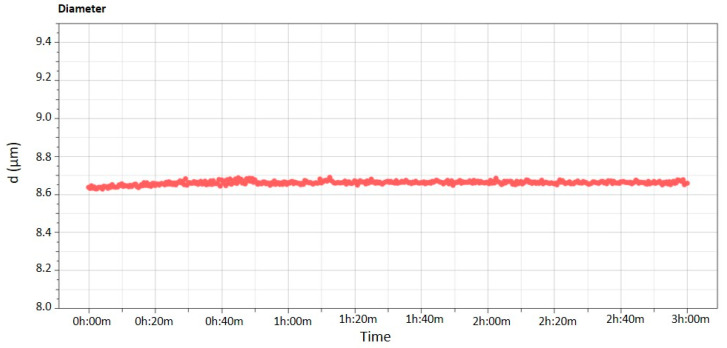
Diameter kinetic profile of multiple emulsion as a function of time (0–3 h). Data are representative of three independent experiments. (The numbers in the axes have been enlarged to improve the visibility of results.)

The long-term stability of multiple emulsions was further supported investigating the mean sizes as a function of the incubation time (3 h). As can be seen in Figure 3 no modifications occurred in size distribution of globules during the Turbiscan analysis. These results were in agreement with previous data obtained from Turbiscan Lab^®^ Expert analysis, thus confirming a great stability of the sample over time. Also simple emulsions reported a constant size distribution during the analysis (Figure S2).

**Figure 4:**
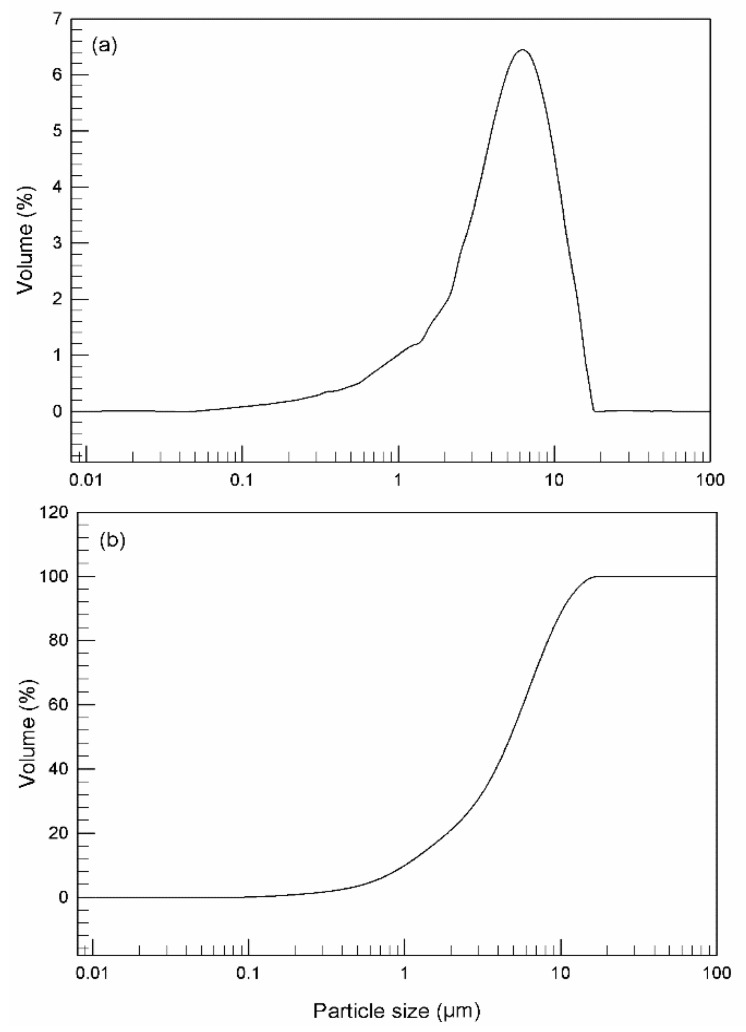
Droplet size distribution curve (Volume %/particle size) (**a**) and cumulative undersizer curve for multiple emulsion; (**b**) obtained by using Mastersizer 2000. The experiments were carried out at 25 ± 1 °C. Data are representative of three independent experiments.

The data previously described for droplets size were confirmed by analysis carried out using a Mastersizer 2000 (Figure 4a). ME droplets exhibited a good homogeneity, as shown in Figure 4b that reports the cumulative undersize curves. The slope of the curve is an index of restricted size distribution and the absence of interferences demonstrates that there is no presence of other size populations.

### 3.2. In Vitro Release and Percutaneous Permeation of Ferulic Acid

The abilities of different kinds of emulsions to control the pharmacokinetic profiles of the delivered drugs have been well investigated and confirmed by several studies [40,41,42]. In this study, the release profile of ferulic acid from multiple emulsions was compared with that recorded from two other simple emulsions. Briefly, ferulic acid was loaded (0.2% *w*/*v*) into delivery systems (O/W, W/O, ME) and the release of the active compound from emulsions and its percutaneous permeation were investigated by using Franz diffusion cells equipped with synthetic membranes or human SCE membranes, respectively.

**Figure 5:**
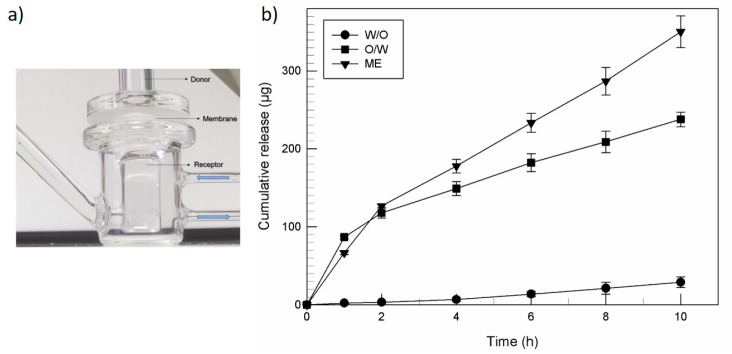
*In vitro* release of ferulic acid in isotonic pH 7.4 phosphate buffer from various emulsion formulations (**b**). Experiments were carried out at 35 °C by using Franz diffusion vertical cells, schematically represented (**a**). Data were expressed as mean values of three experiments ± standard deviations. Standard deviation bars if not visible are within the symbol. *p* < 0.001 for both ME and O/W emulsion, compared to W/O emulsion.

The *in vitro* release experiments (Figure 5) demonstrated that the W/O emulsions had the lowest release of ferulic acid through synthetic membranes, whilst the O/W emulsions showed the fastest and greatest release at 1 h. After 10 h the overall amount of ferulic acid released from ME (~350 µg) was greater than that recorded from O/W emulsion (~237 µg) and both the formulations showed a significantly greater (*p* < 0.001) release, if compared to that obtained from W/O emulsion (~29 µg). Probably, the ferulic acid dispersed into the aqueous phase limits the amount of active readily available for the release, thus not showing a sustained release during time [43,44].

ME have a peculiar release profile, due to their ternary structure, that can modulate pharmaco-kinetic parameters more than other types of emulsions [45]. ME have allowed to obtain an in-time sustained release of the dissolved active compound, due to the ferulic acid dissolved in both internal and external aqueous compartments. More likely, the ferulic acid contained in the external compartment is ready for the early release and diffusion through the membranes, instead the amount contained in the internal compartment ensures a prolonged release up to 10 h. For this reason, a biphasic trend was recorded for this type of emulsion.

**Figure 6:**
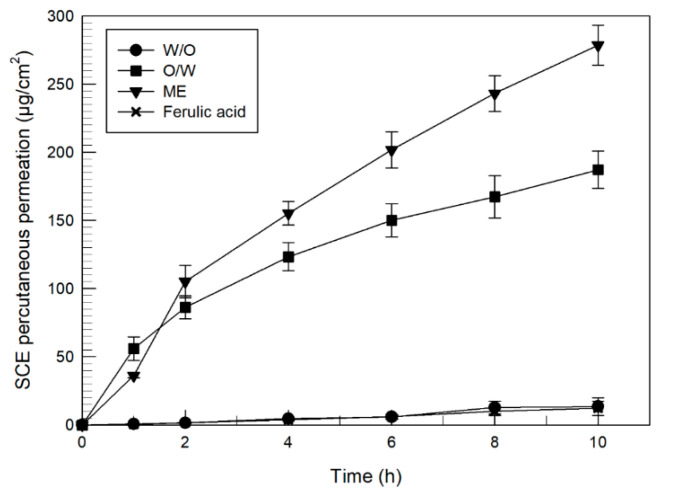
*In vitro* percutaneous permeation of ferulic acid-loaded various emulsion formulations (0.2% *w*/*v*) through SCE membranes. Each value is the mean of six different experiments ± standard deviation. Standard deviation bar if not visible is within the symbol. *p* < 0.001 for both ME and O/W emulsion, compared to the free active. No significative increase was recorded from the permeation profile of ferulic acid-loaded W/O emulsion compared to the free active.

*In vitro* percutaneous permeation experiments were performed using Franz-type cells with human SCE membranes. As can be seen in Figure 6, the recorded data showed the same decreasing permeation order observed for the release studies through synthetic membranes: ME > O/W > W/O. The O/W emulsion was characterized by a quick permeation and at the end of the experiment (10 h) the cumulative amount of the permeated ferulic acid was 187.20 µg/cm^2^ (35.1% of the applied dose, with a flux of 16.98 ± 2.41 µg/cm^2^ h^−1^). The W/O presented the poorest permeation, and after 10 h only 13.5 µg/cm^2^ (2.53% of the applied dose, and a flux of 1.46 ± 0.15 µg/cm^2^ h^−1^) permeated the membrane sheets. It was interesting to note that the permeation curve of W/O emulsion was almost overlapping to that obtained from the permeation of the free active compound and no significative increase in permeation of ferulic acid was obtained in this case.

Finally, ME had the greater percutaneous permeation properties and 278.56 µg/cm^2^ (52.23% of the applied dose with a flux of 27.36 ± 2.46 µg/cm^2^ h^−1^) permeated the skin membranes after 10 h from the administration of sample. The improvement of ME percutaneous permeation could be due to the mixture Brij 72 and Brij 721 that improve the skin hydrating properties and enhance percutaneous permeation of emulsions [27].

### 3.3. In Vivo Studies

Due to its antioxidant properties, the ability of ferulic acid to treat or prevent a physically induced erythema on skin were tested on human volunteers when it was loaded into different kinds of emulsions. Spectral data from reflectance spectrophotometry, can determine the photoprotective effect of these preparations in case of *in vivo* UVB-induced erythema. Erythema index obtained from skin reflectance spectral values was used as a parameter for a good evaluation of skin erythema [33].

**Figure 7:**
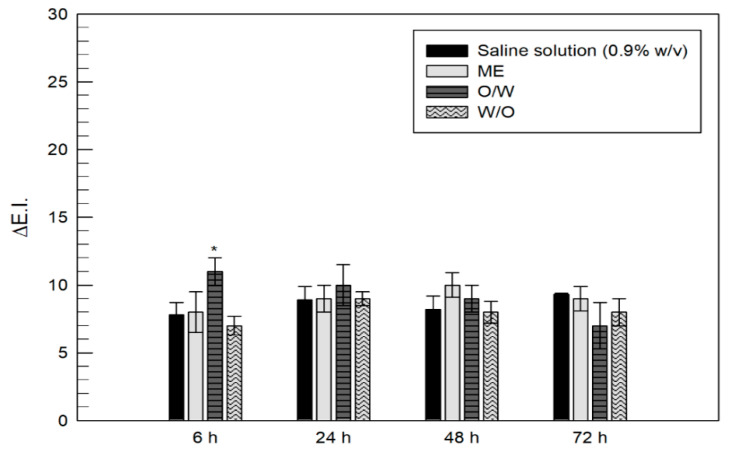
Variation of the Erythema Index (∆E.I.) of skin sites following the skin topical treatment with empty formulations. Data were expressed as mean values of twelve different experiments ± standard deviations. * *p* < 0.05 compared to saline solution.

Before starting the efficacy studies, the *in vivo* tolerability of W/O, O/W and ME was evaluated monitoring the variation of erythema index values (∆E.I.) before and following the cutaneous administration of samples. Experiments were performed on 12 healthy volunteers until 72 h and Figure 7 and Table S1 show the recorded results ± standard deviation. As can be seen in Figure 7, following the administration of ME and W/O emulsion, the obtained ∆E.I. values did not increase significantly compared to values recorded from the administration of saline solution, for all picked time points. In fact, only slight variation (*p* < 0.05), compared to values obtained from the administration of the saline solution were recorded for all exposition timing, thus, showing good safety properties of the analysed samples. Only in the case of O/W emulsion, a significant increase in ∆E.I. values was recorded compared to the administration of saline solution, but it was resolved within 24 h, thereby, showing suitable level of tolerability and safety also for this formulation.

**Figure 8:**
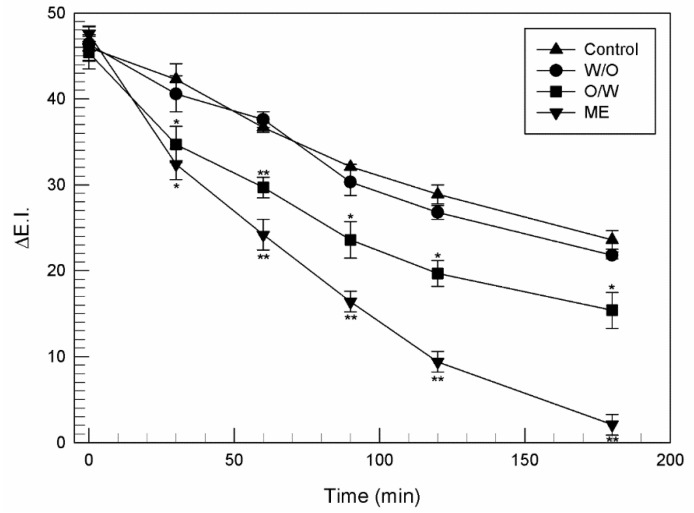
Variation of the Erythema Index (∆E.I.) of skin sites subjected to UVB irradiation and treated with emulsions containing ferulic acid (0.2% *w*/*v*). Data were expressed as mean values of twelve different experiments ± standard deviations. * *p* < 0.05, ** *p* < 0.001 compared to the control.

Further *in vivo* studies have been performed on human volunteers to investigate the photoprotective effect of ferulic acid-loaded emulsions. The erythema was physically induced and the variation of erythema index (ΔE.I.) during the time course has been reported in Figure 8. As can be seen in the figure, after only 120 min from the administration, ME containing ferulic acid, reversed the erythema index at a value similar to the baseline (ΔE.I. = 9.4 ± 1.2), instead volunteer treated with the O/W emulsion still reported great erythema index values (ΔE.I. = 19.7 ± 1.5) at this time. Finally W/O emulsion had a trend not significantly different respect to the untreated control sites during the time course. All these data proved that the ME was the best type of emulsion for the topical administration of ferulic acid and that it was efficacious to treat erythema diseases reducing the excessive production of ROS following the exposition to ultraviolet rays [9].

**Figure 9:**
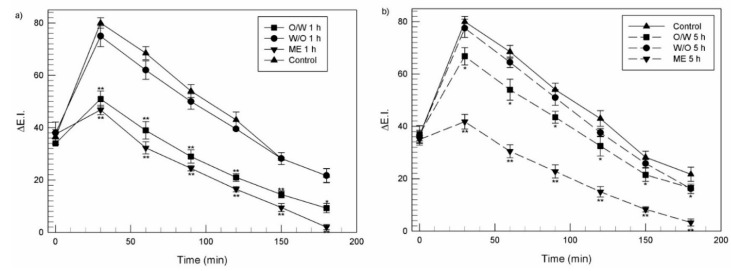
Inhibition of a skin erythema due to the UV-B irradiation elicited by the pretreatment for different time, 1 h (**a**) and 5 h (**b**) of human volunteers with various formulations containing ferulic acid (0.2% *w*/*v*). Results are expressed as a mean value of ∆E.I. (*n* = 12) ± standard deviation as a function of time.

Figure 9 shows the curves of erythema inhibition in the second set of experiments, in which UVB irradiation followed the pre-application of emulsions. Sites pre-treated with O/W and ME promptly antagonized the erythema appearance. Similar profiles of erythema index were recorded after a pretreatment of 1 h with O/W and ME (Figure 9a). However, if skin was pre-treated for 5 h with the same formulations, the efficacy of O/W emulsion to prevent the erythema manifestation was limited. On the contrary, if ME were used for a pre-treatment of skin of 5 h, they showed the best efficacy against the appearance of erythematous states (Figure 9b). These data can confirm the ability of ME to generate a sustained release and a prolonged antioxidant effect of the embedded ferulic acid.

In relation to the W/O emulsion, its topical administration did not show a significative reduction of the induced erythema, if compared to the control, for all the time course, both in the case of 1 h or 5 h of pre-treatment. The not-significant protective effect of W/O emulsion in *in vivo* studies was likely due to the poor ability of the W/O emulsion to release suitable concentrations of ferulic acid and to induce an effective percutaneous permeation of drug, as previously shown in Figure 5, and Figure 6 respectively. A significative reduction compared to the control (*p* < 0.05) has been recorded only after 180 min from a pre-treatment of 5 h, thus, showing similar values compared to a pre-treatment of 5 h with O/W emulsion (Figure 9b).

We can conclude that ME could represent a suitable formulation for the fastest and prolonged release of ferulic acid. FA-loaded ME showed greater antioxidant properties, compared to the control, highlighting interesting photoprotective properties in both the case of pre-treatment or treatment of induced UVB-erythema.

## 4. Conclusions

Ferulic acid, extracted by vegetables and plants, is a potent antioxidant compound that can be used for the treatment of skin damages. However, the great instability of this active compound led to a reduction of its use from skin care industries. Moreover, although ferulic acid is daily introduced with the diet, its skin bioavailability after oral administration is really poor. Taking into account these considerations, it was necessary to realize a system able to protect ferulic acid and to efficiently deliver the active compound through skin. We found that multiple emulsion could be a suitable formulation for the topical percutaneous application of ferulic acid. In detail, our *in vitro* findings showed that multiple emulsions could carry and ensure a sustained release of a large amount of ferulic acid compared to both simple emulsions and the greater efficiency of the multiple emulsion has been confirmed by percutaneous permeation studies. Due to its composition, the multiple emulsion has been shown to induce a greater permeation of ferulic acid than not only the free drug but also the other two preparations (O/W and W/O emulsions). Moreover, the encouraging results obtained through *in vitro* studies were further confirmed by *in vivo* experiments. In particular, in this study, the ability of emulsions to improve the photoprotective activity of ferulic acid was evaluated, and once again, ME was shown to be more effective than simple emulsions, probably due to a more active skin interaction. In detail, the photo-induced erythema on volunteers’ skin was quickly resolved following the administration of ferulic acid-loaded ME, lowering values of erythema index near to zero. Further pre-treatment studies confirmed the great photoprotective ability of ferulic acid-loaded multiple emulsions, in fact, following a 5h pre-treatment with ME, the skin of the volunteers become more protected from a subsequent physical insult and the erythema index remained on lower values than those of the sites pre-treated with the other formulations. The recorded favorable release profile of ferulic acid and *in vivo* efficacy results, make them a versatile carrier. These data suggest multiple emulsions as an innovative and more efficient vehicle for the topical application of several compounds.

## Figures and Tables

**Table 1:** Composition of the W/O/W multiple emulsion (ME), W/O emulsion and O/W emulsion.

Type	Oil Phase	% *w*/*w*	Aqueous Phase	% *w*/*w*
ME	Brij 72	3.00		
	Brij 721	2.00		
	Stearic acid	1.50	Bidistilled water	73.80
	Cetyl alcohol	1.00	Glycerin	4.00
	Arlamol HD	4.00	Preservant	0.20
	Arlamol E	5.00		
	Arlacel P135	0.50		
	Dimethicone	5.00		
W/O	Estol 3603	7.50	Bidistilled water	61.00
	Arlamol HD	15.00		
	Arlamol E	7.50		
	Arlacel P135	4.00		
	Dimethicone	5.00		
O/W	Estol 3603	7.50	Bidistilled water	59.60
	Arlamol HD	7.50	Poloxamer 407	4.00
	Arlamol E	15.00	MgSO_4_·7H_2_O	0.70
	Dimethicone	5.00	Carbomer	0.25
			Triethanolamine	0.25
			Preservant	0.20

## Data Availability

The data presented in this study are available on request from the corresponding author.

## Supplementary Materials

The following are available online at https://www.mdpi.com/2079-4991/11/2/425/s1, Figure S1: photomicrographs of O/W (a) and W/O (b) simple emulsions after 6 h from their preparation. Figure S2. Diameter kinetic profile of O/W (a) and W/O (b) simple emulsions as a function of time (0–3 h). Data are representative of three independent experiments. Table S1. Values of variation of the Erythema Index (∆E.I.) of skin sites following the skin topical treatment with empty formulations. Data were expressed as mean values of twelve different experiments ± standard deviations.
