# Supplementary material for: Improvement of Ferulic Acid Antioxidant Activity by Multiple Emulsions: In Vitro and In Vivo Evaluation
Source: Nanomaterials (Basel). 2021 Feb 8;11(2):425. doi: 10.3390/nano11020425 (PMC7916033; doi:10.3390/nano11020425)
Supplement: Supplementary file 1 [file nanomaterials-11-00425-s001.pdf]

Article

Improvement of Ferulic Acid Antioxidant Activity by Multiple Emulsions: In Vitro and In Vivo Evaluation

Antonia Mancuso [1], Maria Chiara Cristiano [2], Rosanthony Pandolfo [1], Manfredi Greco [3], Massimo Fresta [1] and Donatella Paolino [2,*]

[1] Department of Health Sciences, University "Magna Græcia" of Catanzaro, Campus Universitario "S. Venuta", 88100 Catanzaro, Italy; antonia.mancuso@unicz.it (A.M.); rosantony.pandolfo@unicz.it (R.P.); fresta@unicz.it (M.F.)
[2] Department of Experimental and Clinical Medicine, University "Magna Græcia" of Catanzaro, Campus Universitario "S. Venuta", 88100 Catanzaro, Italy; mchiara.cristiano@unicz.it
[3] Plastic Surgery Unit, Department of Clinical and Experimental Medicine, "Magna Graecia" University, 88100 Catanzaro, Italy; manfredigreco@unicz.it
* Correspondence: paolino@unicz.it; Tel.: +39-0961-3694211

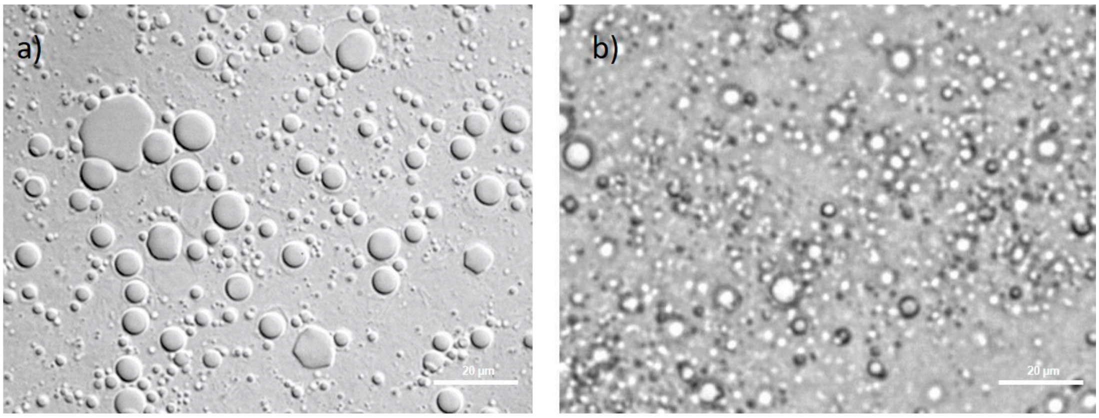

Figure S1. Photomicrographs of O/W (a) and W/O (b) simple emulsions after 6 h from their preparation.

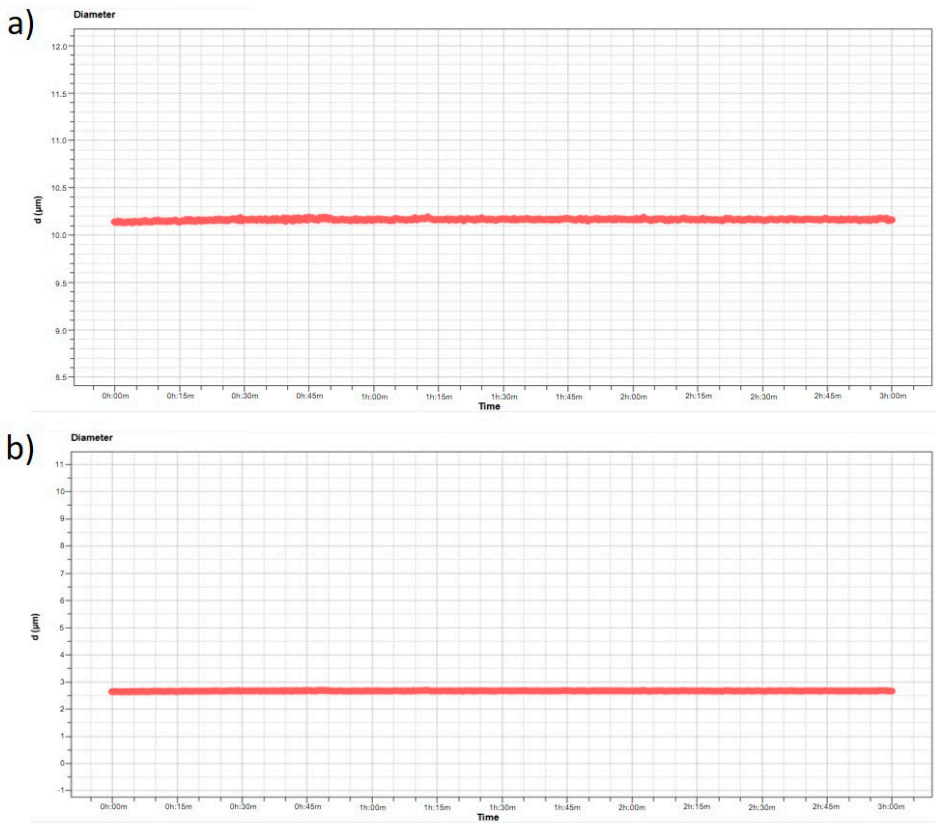

Figure S2. Diameter kinetic profile of O/W (a) and W/O (b) simple emulsions as a function of time (0–3 h). Data are representative of three independent experiments.

Table S1. Values of variation of the Erythema Index (ΔE.I.) of skin sites following the skin topical treatment with empty formulations. Data were expressed as mean values of twelve different experiments ± standard deviations.

Time (h)	Saline solution	ME	O/W	W/O
**6**	7.8 ± 0.9	8.0 ± 1.5	11.0 ± 1.0	7.0 ± 0.7
**24**	8.9 ±1.0	9.0 ± 1.0	10.0 ± 1.5	9.0 ± 0.5
**48**	8.2 ± 1.0	10.0 ± 0.9	9.0 ± 1.0	8.0 ± 0.8
**72**	9.3 ± 0.1	9.0 ± 0.9	7.0 ± 1.7	8.0 ± 1.0
